# Address

**Published:** 1886-01

**Authors:** C. H. James

**Affiliations:** President


### Address.

Delivered before the Ohio State Dental Society, October 29, 1885.

BY DR. C. H. JAMES, PRESIDENT.

FELLOW MEMBERS OF THE OHIO STATE DENTAL SOCIETY :—I am strongly impressed with the idea that my first duty on this occasion, is to express my sincere thanks to you for the honor you have conferred on me, by placing me in the office of President of this Society; an honor, which I assure you, is highly appreciated, and which I sincerely hope I shall never forfeit.

It will be my object and desire to so conduct the business pertaining to this office as to leave no regret on your part that you placed me here, and with the satisfaction on my part, of yielding to my successor, having received the verdict: "Well done, good and faithful servant."

Owing to the peculiar circumstances surrounding this meeting, I deem it advisable to deviate from the usual custom, and instead of, at my retirement, calling your attention for a brief period to some professional topic, at this time, to ask your indulgence for a little while, to the matter we have in hand, viz: a thorough reorganization of this Society; and, in so doing, pardon me if I attempt to refresh your memories with a little history.

May 21st, 1866, was issued the following appeal to the members of our Profession, in this State:

"The entire Dental Profession of the State of Ohio, are hereby cordially invited to meet in mass convention in the City of Columbus, at Nauton's Hall, on Tuesday and Wednesday, the 26th and 27th of June, to form a State Dental Society, and to devise and adopt such other measures as tend to elevate and advance the interests of the Profession. Come up, brethren and let us have such a meeting as our Profession has not yet seen. The advantages to be derived from such an organization cannot be over-estimated. Other States around us are fully organized. Shall Ohio lag behind?"

This appeal was enthusiastically responded to, and on the 26th of June, an organization was effected, and the Society formed with a roll of forty-eight members, eight of whose names appear on the roll of this reorganized Society. The next year, 1867, two meetings were held, one in June and one in December, after that come annual sessions with unabating interest, and increasing membership until 222 names appeared on our roll. We should congratulate ourselves that much good was accomplished by the old association, and its disolution was only made necessary by a too close application to legitimate business, and an entire oversight of the fact that in the mutations of time we did not keep in view the necessity for changes in our Constitution and By-Laws, by which disturbing elements could be excluded, and questions having a tendency to detract from the main objects, could be avoided.

It is hoped our committee on Constitution and By-Laws will not burden us with an excess of laws, but submit a report at once, simple and brief, but at the same time, comprehensive, and capable of meeting every emergency.

It would seem like carrying coals to Newcastle, to argue at this late day, in favor of associated effort, both for the good of the profession and its individual members; but there are so many who take no interest outside of themselves or their offices, leaving the profession to the care of a few; that something should be done to awaken the proper interest, and a few must for a time, at least, in the future as in the past, bear the burden.

Let one object of our Association be to lessen the labors of these few faithful servants, by dividing the burden with the many, and make them all willing workers instead of indifferent approvers.

I cannot resist the temptation at this time, of making a few remarks upon the advantages such an Association as this bids fair to be, has upon the individual.

He who scorns association, necessarily places himself beyond the reach of progress, and content with his superficial knowledge, moves in a rut, or is guided as the train upon a fixed tract that does not allow a movement either to the right or left, and when traveled much reveals nought but old and familiar scenes, which add no new beauties, and awaken no new desires or ambitions; monotony dulls energy, and results in an indifferent discharge of duty, to no credit and but little profit.

He who contents himself with seclusion, ignoring companionship with the members of his profession, necessity becomes disgusted with himself, his neighbors, and his calling. He never feels that intense satisfaction of having gloried in the utterance of a new idea or method, and never rejoices in a union of hearts, hands, thoughts and triumphs, afforded by association.

He shuts himself up within himself, seeing self encouraging selfishness, suspicion, distrust, and envy, until all better feelings are blunted or entirely destroyed, and all others in the profession, viewed from his stand-point, are precisely his counterpart.

This picture is exactly the reverse of the objects of our Society.

By mutual exchange of thoughts and ideas and methods of practice, we elevate ourselves and associates, to the same glorious and magnanimous position of working out our profession's good; by Association, we gain invigoration and enthusiasm, we have a kindly interest in one another and feel bound as in a common cause.

By uniting forces of themselves ineffectual, we produce powerful results not otherwise obtained. Through our Societies our profession's honor is always fostered or preserved, each member becomes his brother's keeper, ready to chide, advise, encourage, and if need be condemn, and as man is by nature a social being, it seems most fitting that he should ally himself at least with those of his profession or calling, for purposes of advancement, which cannot be otherwise than beneficial.

We will not pursue this idea further for fear of encroaching upon the first subject in our order of business, which we have no doubt, will be ably presented and fully discussed.

We have faith that those of us who have stood shoulder to shoulder as it were, since 1866, will fully appreciate the situation, and work out the problem of reorganization to a successful issue, and having learned experience by the past, lay deep and broad the foundation upon which to build a superstructure that will endure for all time, an everlasting monument to the memory of its founders, and to which all future members of our profession may look with increasing zeal, determined to perpetuate its grandeur and magnificence, and hand it down untarnished in its glorious proportions from generation to generation.

One of our duties at this meeting will be the formulation of a bill regulating the practice of dentistry in this State which will be effective, and a substitute for the one which now adorns our statutes without accomplishing any good for want of an executive clause.

What is everybody's business is nobody's, and this saying has been abundantly proven in this case. Another duty is the framing and adoption of a Constitution and By-Laws suited to our age of progress, and that will be as the mariner's chart to mark out the rocks and shoals which were encountered in our old Society, and come near causing a disastrous wreck.

These two indispensable duties need not consume much time, as the Committee on State Law is doubtless fully prepared to present a report that will meet your hearty approval, and I am informed, a Constitution and By-Laws will be prepared with so much care, that there will be little to do except to approve.

Let each one of us labor earnestly for the consummation of these two objects, setting aside, if necessary, unimportant personal preferences for the ultimate honor and good of all.

